# Evidence on global medical travel

**DOI:** 10.2471/BLT.14.146027

**Published:** 2015-09-18

**Authors:** Kai Ruggeri, Ladislav Záliš, Christopher R Meurice, Ian Hilton, Terry-Lisa Ly, Zorana Zupan, Saba Hinrichs

**Affiliations:** a Department of Psychology, University of Cambridge, Downing Street, Cambridge, CB2 3EB, England.; b Department of Psychology, Masaryk University, Brno, Czech Republic.; c North Central College, Naperville, Illinois, United States of America.; d Medical Faculty, University of Köln, Köln, Germany.; e Department of Psychology, University of Warwick, Coventry, England.; f The Policy Institute, King’s College London, London, England.

## Abstract

The potential benefits of travelling across national borders to obtain medical treatment include improved care, decreased costs and reduced waiting times. However, medical travel involves additional risks, compared to obtaining treatment domestically. We review the publicly-available evidence on medical travel. We suggest that medical travel needs to be understood in terms of its potential risks and benefits so that it can be evaluated against alternatives by patients who are seeking care. We propose three domains –quality standards, informed decision-making, economic and legal protection – in which better evidence could support the development of medical travel policies.

## Résumé

Les avantages potentiels de la traversée de frontières nationales pour recevoir un traitement médical résident notamment dans une meilleure prise en charge, des coûts moindres et des temps d'attente réduits. Il est cependant plus risqué de voyager pour bénéficier de soins médicaux que de recevoir un traitement à l'intérieur du pays. Nous avons analysé les données scientifiques publiquement disponibles concernant le tourisme médical. Il nous apparaît essentiel de considérer le tourisme médical en fonction de ses risques et de ses avantages potentiels afin que les patients qui nécessitent des soins puissent le comparer aux autres solutions possibles. Nous estimons que l'amélioration de la qualité des données scientifiques dans trois domaines – normes de qualité, prise de décision éclairée et protection économique et juridique – favoriserait l'élaboration de politiques relatives au tourisme médical.

## Resumen

Los posibles beneficios de cruzar las fronteras nacionales para obtener tratamientos médicos incluyen una mejora de la atención, una disminución de los costes y una reducción de los tiempos de espera. Sin embargo, el turismo sanitario implica riesgos adicionales en comparación con obtener el tratamiento en el país. Se revisaron los datos públicos sobre el turismo sanitario. Nuestra sugerencia es que el turismo sanitario se entienda en términos de sus posibles riesgos y beneficios, de modo que aquellos pacientes que busquen atención médica puedan evaluarlos frente a otras alternativas. Proponemos tres ámbitos (normas de calidad, toma de decisiones informada y protección económica y legal) en los cuales unos datos más claros podrían apoyar el desarrollo de las políticas para el turismo sanitario.

## ملخص

تشمل المنافع المحتملة للسفر عبر حدود الدول للحصول على العلاج الطبي خدمات الرعاية الصحية المحسَّنة، والتكاليف المخفضة، وفترات انتظار أقصر. وعلى الرغم من ذلك، تتضمن السياحة لأغراض طبية بعض المخاطر الإضافية مقارنةً بالحصول على العلاج داخل الدول. وقد أجرينا مراجعة للأدلة المتاحة على المستوى الحكومي فيما يتعلق بالسياحة لأغراض طبية. ونشير إلى الحاجة إلى فهم السياحة لأغراض طبية في إطار المخاطر والمنافع المحتملة لها، ومن ثم يمكن للمرضى الباحثين عن خدمات الرعاية الصحية تقييمها بالمقارنة بغيرها من البدائل. كما نطرح ثلاثة محاور – وهي معايير الجودة، واتخاذ القرارات المستندة إلى المعلومات، والحماية القانونية والاقتصادية – والتي يمكن من خلالها دعم وضع سياسات السياحة لأغراض طبية اعتمادًا على الأدلة.

## 摘要

跨国医疗旅游的潜在好处包括获得更加优质的护理服务、更低的花费和更短的等待时间。但是，与在国内就医相比，医疗旅游存在一些额外的风险。通过审查现有医疗旅游的证据，我们建议寻求医疗护理的患者全面了解医疗旅游的潜在风险和好处，以便与其他医疗方式进行对比评估。我们提议从质量标准、科学的决策制定、经济和法律保护三个方面入手，这三方面的良好证据能够为医疗旅游政策的制定提供支持。

## Резюме

Потенциальные преимущества поездки за границу с целью получения лечения включают лучший уход, снижение затрат и меньшее время ожидания лечения. Однако медицинский туризм подразумевает и дополнительные риски в сравнении с лечением дома. Нами были рассмотрены имеющиеся в общественном доступе свидетельства относительно поездок с медицинскими целями. Мы предлагаем рассмотреть медицинский туризм с точки зрения риска и пользы, чтобы пациенты, нуждающиеся в лечении, могли оценить их и сравнить с альтернативными вариантами. Мы предполагаем три области оценки: стандарты качества, принятие информированного решения, экономическую и юридическую защиту, — в которых наличие лучших свидетельств послужит поддержкой при разработке принципов медицинского туризма.

## Introduction

Medical travel is projected to expand globally in the next decade.^1^^,^^2^ Citizens in the United States of America, for instance, already receive significant volumes of services abroad, both for urgent and elective procedures.^3^ The growth in medical travel is largely due to improved availability of health technology, decreasing costs for travel and advertising by companies wishing to attract patients.^4^^,^^5^ Medical tourism has been described as “travel across international borders with the intention of receiving some form of medical treatment. This treatment may span the full range of medical services, but most commonly includes dental care, cosmetic surgery, elective surgery and fertility treatment.”^6^

Some authors distinguish between medical tourism (travel for wellness, cosmetic or other non-essential procedures) and medical travel (travel with the purpose of receiving treatment that, in the opinion of a health professional, is essential to maintain quality of life). For the purposes of this paper, we define medical travel as including both essential and non-essential treatment. Although it is clear that cost, accessibility and quality are the main motivating factors for medical travel, data are largely limited to anecdotal projects from the perspective of a single country.^3^^,^^7^^–^^12^ While some data on transnational health-care practices are available,^10^^,^^13^ current data on the outcomes of medical travel are insufficient and rarely generated using rigorous methods.^14^

The potential benefits of medical travel include improved care, decreased costs and reduced waiting times.^15^ Medical travel may increase access to certain treatments for local communities through improved infrastructure and higher demand. In India, for example, a two-tiered approach employed in some hospitals resulted in improved services for local patients and tailored services for medical travellers.^7^^,^^16^ Panama and Thailand have also developed services that were initially intended to attract foreign patients but also resulted in new facilities available for locals.^16^ A competitive market for health care could help to control medical spending and increase access by introducing patients to new locations and lower costs for care. For developed countries, medical travel may result in small reductions in national health costs.^17^

Medical travel involves additional risks, compared to obtaining treatment domestically. If complications or adverse outcomes occur, additional expenses are likely, but insurance companies may not be willing to cover these costs.^18^^,^^19^ Medical travel may jeopardize the well-being of vulnerable individuals who are ill, in unfamiliar locations or cultures and who lack social support. Factors such as social support, familiarity with language and carers and proximity to home can be important for recovery from clinical procedures.^20^^,^^21^ Medical travel often involves travel from high-income to low- or middle-income countries,^5^^,^^22^ with different standards of clinical practice.

The growth in medical travel has implications for health service provision in destination countries. Access to health care for local residents might be adversely affected if local health professionals devote their time to treatment of foreigners rather than local communities. The ethical issues raised by medical travel are particularly acute in countries where access to basic medical care may be unaffordable for the local population. Here we review the scientific literature relevant to development of global policy on medical travel. Medical travel needs to be understood in terms of its potential risks and benefits so that it can be evaluated against alternatives.

## Literature search

We carried out a non-systematic literature review. Articles were considered if they were found in any of six databases under the terms “medical tourism” or “medical travel” since the year 2000, with no language restrictions. The databases used were PubMed, EconLit, Google Scholar, the World Bank research database, Europe PubMed Central and EMBASE. We identified primary themes to be reviewed through common arguments used in higher quality sources as well as through common topics in low-quality or potentially very biased material. Papers were included if they provided specific data, policy or practice analyses, or substantial insight to key themes, regarding travel for necessary care. Where available, we extracted estimates of the numbers of medical travellers within the last 10 years.

## Available data

Most of the available data on medical travel is of poor quality. Sources are often not accessible or do not explain how estimated figures were calculated.^10^ There are variations in the definition of medical travellers and a lack of agreed methods for data collection.^23^^,^^24^ For example, Singapore collects the data on medical travel using exit polls at the airport, including only travellers who had arrived with a stated primary purpose of obtaining medical care. Thailand counts the number of foreigners obtaining medical care at hospitals, including foreign travellers who did not travel primarily to obtain health care. Estimates are framed as medical travel, potentially including visitors coming for beauty treatments using spa and wellness resorts. These conflate cosmetic and other elective procedures with essential treatments. For example, Hungary is often portrayed as one of the hubs of medical travel in Europe with up to 1.8 million medical travellers annually, yet the majority of those are day visitors on wellness trips or travelling for dental care.^25^^,^^26^ Thailand reports over 1.5 million medical travellers annually, but it is estimated that only a third travelled specifically for medical treatment.^27^

The difference between high and low estimates of medical travellers increases if a wider range of timelines, methods and measures are included.^28^ Without official national data, numbers presented by some countries and hospitals may be exaggerated, potentially for the purpose of implying growth and success and encouraging private sector investment and national support.^23^

Table 1 presents numbers of medical travellers as reported in papers that we reviewed. Considering the limitations of the sources, this table cannot accurately reflect the amount and structure of current medical travel, however it does give a basic overview of what is generally reported by researchers. This table reflects publicly-available data and summarizes the kind of information researchers, policy-makers and health-care professionals may use when making decisions. The data available for different countries and regions indicate a prominent focus on Asian countries, including India, Malaysia, Singapore and Thailand. Additional data, based on narrative and speculative evidence were not included in the table. It does not include reports that required a paid subscription to be accessed.

**Table 1:** Reported estimates of medical travellers to receiving countries

Receiving country	Estimated no. of annual medical travellers	Year and reference
Australia	13 000	2010^29^
Brazil	49 000–180 000	2005^17^ and 2009^30^
Costa Rica	25 000–150 000	2006,^31^ 2007^32^ and 2008^33^
Cuba	3500	2003^26^
Cuba	200 000	2007^32^
Egypt	68 000–108 000	2003,^34^ 2004,^28^ 2005^28^ and 2006^35^
Germany	50 000–70 000	2008^28^ and 2009^29^
Hungary	1 500 000–1 800 000	2007^28^ and 2009^27^
Hungary	300 000	2008^30^
India	1 000 000–1 180 000	2004^36^ and 2005^37^
India	100 000–150 000	2005^24^^,^^29^^,^^38^^–^^40^
India	300 000–731 000	2006,^41^ 2007,^1^ 2008^5^ and 2010^39^
Israel	35 000	2009^32^
Jordan	120 000–250 000	2002,^34^ 2004^28^ and 2009^30^
Malaysia	300 000–489 000	2006,^41^ 2007,^24^^,^^29^^,^^42^ 2008^5^ and 2010^39^
Philippines	100 000–250 000	2006,^24^^,^^41^ 2009^2^ and 2010^43^
Republic of Korea	60 000	2009^2^^,^^29^
Singapore	270 000–450 000	2004,^31^ 2005,^24^^,^^40^ 2006^1^^,^^29^ and 2008^5^
Singapore	571 000–725 000	2007,^42^ 2008^2^ and 2010^39^
South Africa	330 000	2010^44^
Thailand	450 000–700 000	2004,^45^ 2006^41^ and 2007^25^
Thailand	1 000 000–1 580 000	2004,^36^^,^^38^ 2006,^46^ 2007,^1^ 2008^5^^,^^29^ and 2010^39^
Tunisia	10 000–42 000	2002,^34^ 2003^38^ and 2007^28^
Turkey	15 000	2007^28^
United Kingdom	52 000	2010^47^
United States of America	250 000–400 000	2006^16^ and 2007^1^^,^^5^

Note: Reports were identified by a non-systematic literature review of PubMed, EconLit, Google Scholar, the World Bank research database, Europe PubMed Central and EMBASE.

Clear parameters are needed for future work on medical travel, to develop an evidence base for policy development. The existing literature on medical travel emphasizes economic interests.^6^^,^^17^^,^^23^^,^^48^ Below, we discuss international quality standards, the motivation of medical travellers and the legal and economic situation relevant to medical travel.

### International quality standards

While many different types of national quality-control measures exist, they are not applicable across international borders and this increases the risk of uninformed decision-making by potential medical travellers.^49^ The lack of international standards further increases the risk for patients and their local care providers as complications may arise after medical travellers return home.^10^^,^^18^^,^^19^ To establish international quality standards, definitions and comparable indicators should be established, with oversight from a major international body.

### Patient decision-making

More data on patient decision-making needs to be collected to ensure the practical value of any evidence generated on medical travel. Existing patient choice models indicate that there are a large number of considerations when deciding where to receive care and highly specified measures are needed to evaluate this at a global level.^10^^,^^32^^,^^50^ The drivers and barriers that precede medical travel need to be assessed beyond general economic and availability factors.

Knowledge of factors involved in patient choices about cross-border care is sparse.^10^^,^^17^ Although evidence is starting to emerge,^10^ there is an overall deficiency in the literature as to why potential patients travel to specific locations for medical treatment. Future research could build on national studies, which have clearly indicated that cost, quality and availability are critical in the decision-making process.^51^ Such evidence would make a significant contribution, particularly if coinciding with better mapping of the flow of medical travellers.^6^^,^^7^ Finally, if patients, carers, insurers and other stakeholders are to be expected to make informed decisions, responsibility for costs needs to be clarified. Understanding the cultural factors that influence patients’ decisions will provide essential background for the development of medical travel policies.^51^

### Frameworks

To prevent harm, legal and economic frameworks for medical travel are needed.^6^^,^^52^ Existing governance structures and legal frameworks on treatment and care standards need to be harmonized and international quality standards need to be enforced and maintained. The current lack of regulation in medical travel creates risks for patients due to a lack of oversight and variable standards of practice.^33^ Economic protection through regulated insurance for patients as well as through local regulation (to avoid negative effects on access to care for local residents) must be in place. This regulation will be critical as some health systems face increased demand from an international market that may result in unwanted increases in costs of care for locals.^53^

Legal frameworks need to clarify liabilities for adverse events and ensure equality of access for the local population. Access to health care has implications beyond individual care, including the perception of health-care system quality, which in turn influences individual well-being.^54^ Evaluations of medical travel should measure the impact on access to care for local communities, to ensure they are not negatively affected by such changes.^55^

## Conclusion

There are considerable gaps in the current literature concerning the extent to which international health services are consumed and the needs of medical travellers are met. We suggest three key domains (quality standards, informed decision-making, economic and legal protection) for which scientific evidence would support the development of medical travel policies. There are challenges to obtaining such data, including barriers to accessing up-to-date information in governmental records, in health systems reviews, and in confidential databases kept by insurance firms. It is also difficult to find representative samples of patients who travel for care. There is currently a lack of consistent data on the specific procedures these patients seek, how many patients are involved and how much expenditure occurs. Effective research in medical travel as a global phenomenon requires consideration of all three domains, with the overall goal of improving access, quality of care, and health equity.
